# Association between atherogenic index of plasma and type 2 diabetic complications: a cross-sectional study

**DOI:** 10.3389/fendo.2025.1537303

**Published:** 2025-02-04

**Authors:** Yue-Yang Zhang, Xiao-Yu Yang, Qin Wan

**Affiliations:** ^1^ Department of Endocrinology and Metabolism, Affiliated Hospital of Southwest Medical University, Luzhou, China; ^2^ Metabolic Vascular Disease Key Laboratory of Sichuan Province, Luzhou, China; ^3^ Sichuan Clinical Research Center for Diabetes and Metabolism, Luzhou, China; ^4^ Sichuan Clinical Research Center for Nephropathy, Luzhou, China; ^5^ Cardiovascular and Metabolic Diseases Key Laboratory of Luzhou, Luzhou, China

**Keywords:** atherogenic index of plasma, type 2 diabetes, diabetic complications, metabolic management center, cross-sectional study

## Abstract

**Background:**

The Atherogenic Index of Plasma (AIP) was originally developed primarily as a marker for assessing atherosclerosis. Consequently, this study investigates the potential association between AIP and type 2 diabetic complications through a cross-sectional design.

**Methods:**

The National Metabolic Management Center(MMC) serves as a comprehensive platform dedicated to the establishment of standardized protocols for the diagnosis, treatment, and long-term follow-up of metabolic diseases. Following the relevant inclusion and exclusion criteria, a total of 3,094 patients were enrolled for subsequent analysis. In this study, logistic regression, restricted cubic splines, and subgroup analyses were employed to evaluate the association between the AIP and four major complications of type 2 diabetes, namely, type 2 diabetes with carotid atherosclerosis (DA), diabetic kidney disease (DKD), diabetic retinopathy (DR), and diabetic peripheral neuropathy (DPN).

**Results:**

The logistic regression results demonstrate that in the fully adjusted model, each SD increase in AIP correlates with an elevated risk of type 2 diabetic kidney disease (DKD), with the risk of kidney damage intensifying alongside higher AIP groupings. The RCS analysis and subgroup analyses similarly revealed a dose-response relationship between AIP levels and the risk of DKD. Furthermore, the AIP was not found to be statistically significantly associated with DA, DR,and DPN.

**Conclusions:**

The AIP may serve as a valuable predictive indicator for evaluating kidney damage in patients with type 2 diabetes, and regular screening of AIP in this population could provide significant benefits in the prevention of DKD.

## Highlights

- Systemic damage associated with type 2 diabetes is a major cause of disability and even death in people with type 2 diabetes;
- AIP is currently used mainly for the prediction of atherosclerosis;
- AIP has a potential dose-response association with DKD and can be considered as a potential biomarker for the prediction of DKD.

## Introduction

It is well known that type 2 diabetes is a serious chronic disease characterized by elevated blood glucose levels due to relative or absolute insulin deficiency, and it is considered an important component of endocrine metabolic disorders (1, 2). The 2021 Diabetes Atlas published by the International Diabetes Federation (IDF) indicates that the number of diabetes patients will reach 643 million by 2030 and is expected to rise to an astonishing 783 million by 2045, with global healthcare expenditures related to diabetes potentially exceeding $1.05 trillion (3, 4). The Global Burden of Disease Study 2021 indicates that as of 2021, diabetes has become the eighth leading risk factor for individual death and disability (5).

Indeed, numerous studies investigating the causes of diabetes-related mortality have demonstrated that the majority of individuals with type 2 diabetes present with at least one comorbid systemic complication, including neuropathy, nephropathy, retinopathy, and notably, cardiovascular damage, which constitutes a principal cause of mortality among diabetes patients (6, 7). Research indicates that an increasing number of individuals with type 2 diabetes are being diagnosed at a younger age (under 40 years), leading to diminished life expectancy and an increased number of years of life lost (8). Consequently, the American Diabetes Association has consistently underscored in its standards of diabetes care the critical importance of implementing appropriate strategies to prevent and delay diabetes-related multisystem complications (9).

Although glucose metabolism and lipid metabolism are relatively independent metabolic pathways, they are intricately interconnected through the renin-angiotensin-aldosterone system, mitochondrial function, oxidative stress, and inflammatory responses. These disrupted molecular and cellular mechanisms collectively contribute to the development of diabetes and atherosclerosis (10). As the global burden of metabolic cardiovascular diseases escalates, an increasing number of studies underscore the necessity for critical preventive and therapeutic interventions to mitigate the impact of metabolic factors on cardiovascular health (11). Peripheral neuropathy represents one of the most prevalent, complex, and debilitating complications among diabetic patients, significantly heightening the risk of ulcers, non-traumatic amputations, and foot infections, which may result in long-term disability and impose considerable economic and psychological burdens on individuals with type 2 diabetes (12). Furthermore, an observational study conducted in an Asian population revealed that the prevalence of type 2 diabetic kidney disease (DKD) among individuals with type 2 diabetes is as high as 58.6%, with DKD recognized as a major contributor to chronic kidney disease and end-stage renal disease (13).

Prior studies have identified clinical biomarkers linked to type 2 diabetic complications, including mannose, glycerol, alanine, and the triglyceride-glucose index (14). Our previous research has also identified associations between indicators of kidney function, thyroid hormones, and type 2 diabetic complications (15). The atherogenic index of plasma (AIP), introduced by Dobi et al. (16) in 2001 as a predictor of atherosclerosis, is derived from the logarithmic transformation of the ratio of triglycerides to high-density lipoprotein. Subsequent studies conducted by numerous researchers have demonstrated that AIP is positively correlated with cholesterol esterification rates, lipoprotein particle size, and residual lipoprotein levels. Thus, AIP is regarded as a reliable indicator for predicting atherosclerosis and cardiovascular diseases (17, 18). Furthermore, a population-based study identified that the AIP index may serve as an early marker for chronic kidney disease and liver injury in patients with type 2 diabetes (19).

Nevertheless, research on the association between AIP and type 2 diabetic complications remains limited. Consequently, this study seeks to evaluate the potential association between AIP and type 2 diabetic complications utilizing data from the National Metabolic Management Center (MMC).

## Method

### Study design and population

Established in 2016, the MMC aims to establish a standardized platform for the diagnosis, treatment, and long-term follow-up of metabolic diseases, encompassing nearly 300 hospitals nationwide (20). The study protocol and informed consent documents received approval from the Institutional Review Board of Ruijin Hospital, affiliated with Shanghai Jiao Tong University School of Medicine. All participants provided written informed consent prior to their participation in the study.

**Figure 1:**
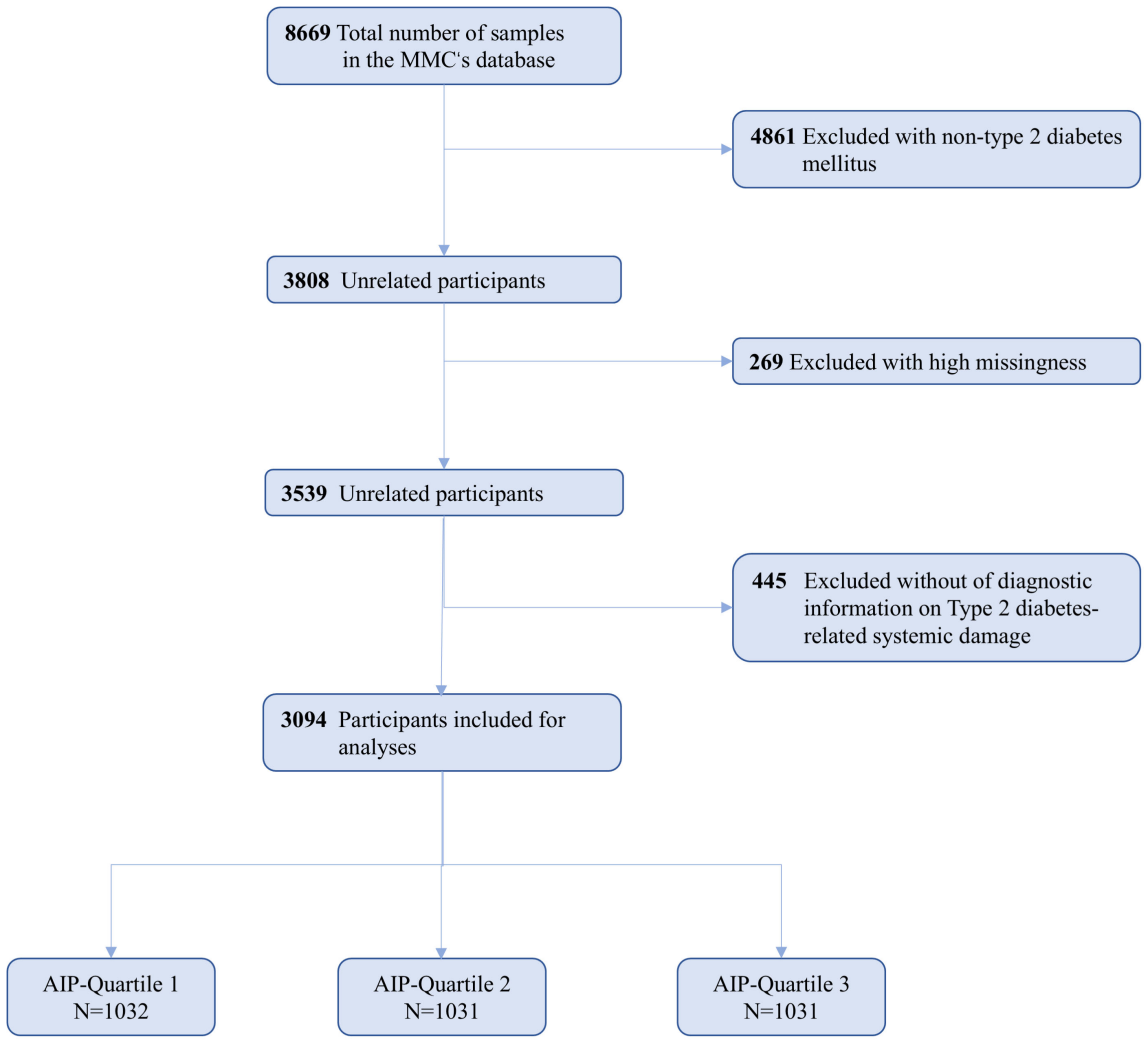
Flowchart for the Selection of the Analyzed Study Sample From the MMC’s database.

The MMC project encompasses several provincial sub-centers, each representing distinct regions, with standardized research protocols and methodologies implemented uniformly across all centers. This study primarily utilized data from the MMC Sichuan sub-center. This study initially screened 8,669 patients who were hospitalized for the first time between 2017 and 2023 at the Sichuan Provincial Center of the MMC. Initially, 4,861 patients without a diagnosis of type 2 diabetes were excluded from the analysis. Furthermore, 269 participants with a high baseline data missing rate and 445 participants who did not meet the diagnostic criteria for type 2 diabetic complications were excluded from the analysis as well. Ultimately, a total of 3,094 participants were included in the final analysis, as illustrated in **Figure 1**.

### Data collection

Demographic and anthropometric data for all participants, including gender, age, height, weight, waist circumference, and blood pressure, were extracted using the MMC-specific electronic medical record system. Smoking status was classified into current smokers and non-smokers; current smokers were defined as individuals who smoked at least 7 cigarettes per week for a minimum of 6 months, while non-smokers were defined as those who either had previously smoked under the same criteria but had since quit or who had never smoked. Drinkers were categorized into current drinkers and non-drinkers; current drinkers were defined as individuals who consumed alcohol at least once per week for a minimum of 6 months, while non-drinkers were defined as those who had previously consumed alcohol under the same criteria but had since quit or who had never consumed alcohol. Laboratory test indicators were collected by trained nursing personnel, who obtained fasting blood samples (requiring a fasting period of at least 8-12 hours) from the patients and subsequently sent these samples to the central laboratory for blood metabolite analysis. Glycated hemoglobin levels in capillary whole blood were measured using high-performance liquid chromatography (VARIANT II system; Bio-Rad, Hercules, CA), whereas lipid levels were assessed using an automated analyzer (Abbott Laboratories, Abbott Park, IL). Additionally, all patients were stratified into three groups based on the tertiles of AIP: Q1 (AIP < 0.07, N = 1032), Q2 (AIP: 0.07-0.36, N = 1031), and Q3 (AIP > 0.36, N = 1031).

### Ascertainment of covariates

Drawing from prior research experience, we developed multiple regression models by incorporating a range of confounding factors (21, 22). Demographic information encompassed age, sex (classified as “male” and “female”), waist circumference(WC), body mass index (BMI), history of hypertension (designated as “yes” or “no”), smoking status (designated as “yes” or “no”), and alcohol consumption status (designated as “yes” or “no”). Laboratory test indicators encompassed systolic blood pressure (SBP), diastolic blood pressure (DBP), fasting blood glucose (FBG), triglycerides (TG), serum creatinine (Cr), total cholesterol (TC), high-density lipoprotein cholesterol (HDL-C), low-density lipoprotein cholesterol (LDL-C), and glycated hemoglobin (HbA1c).

### Assessment of type 2 diabetes and type 2 diabetes-related complications

The diagnosis of type 2 diabetes is based on the latest guidelines from the American Diabetes Association (ADA) 2024, with at least one of the following criteria met: 1) Fasting blood glucose level ≥ 7.0 mmol/L; 2) Postprandial blood glucose level ≥ 11.1 mmol/L two hours after an oral glucose tolerance test (OGTT); 3) Glycated hemoglobin level ≥ 6.5%; 4) Random blood glucose ≥ 11.1 mmol/L accompanied by other classic symptoms of diabetes (23). Type 2 diabetes complications are diagnosed according to the International Classification of Diseases (ICD-10) codes, which encompass type 2 diabetes with carotid atherosclerosis (DA, I70.806), diagnosed via B-mode ultrasound showing carotid intima-media thickness ≥1 mm or the presence of atherosclerotic plaques in the carotid arteries; diabetic kidney disease (DKD, E11.2), diagnosed based on a random urinary albumin-to-creatinine ratio (UACR) ≥30 mg/g or urinary albumin excretion rate (UAER) ≥30 mg/24h, with repeat testing within 3 to 6 months, and at least two of three subsequent UACR or UAER tests exceeding the threshold; diabetic retinopathy (DR, E11.3), confirmed through lesions assessed by a specialist ophthalmologist using fundus photography; and diabetic peripheral neuropathy (DPN, E11.4), identified by abnormalities in the peroneal nerve alongside at least one other nerve abnormality detected on nerve conduction studies (24–27).

### Statistical analysis

Appropriate statistical methodologies were employed to systematically characterize the baseline characteristics of participants based on different types of data. Continuous variables are presented as means ± standard deviations, whereas categorical variables are reported as counts (percentages). For normally distributed data, analysis of variance (ANOVA) was employed to compare mean differences between groups, while the Kruskal-Wallis test was utilized for skewed data, and the chi-square test was applied to categorical variables. Logistic regression was employed to investigate the potential association between the AIP and systemic complications associated with type 2 diabetes, estimating odds ratios (OR) and 95% confidence intervals (CI) across three distinct models. Model 1 was adjusted for age and gender. Model 2 was adjusted for age, gender, WC, BMI, DBP, SBP, LDL-C, TC, Cr, FBG, and HbA1c. Model 3 offered comprehensive adjustments for age, gender, WC, BMI, DBP, SBP, LDL-C, TC, Cr, FBG, HbA1c, smoking status, alcohol consumption, and history of hypertension. The trend p-value was estimated by treating the groups delineated by AIP quartiles as a continuous variable. Additionally, a restricted cubic spline (RCS) with five knots was employed to examine the nonlinear association between AIP and systemic complications associated with type 2 diabetes. Finally, subgroup analyses were performed for age, gender, BMI, and history of hypertension to explore potential interactions between confounding factors and AIP, including age (<60, ≥60), gender (male, female), BMI (<25, ≥25), and history of hypertension (Yes, No). To maintain consistency in statistical evaluations, all subgroup analyses were conducted using logistic regression analysis based on Model 3. Furthermore, non-significant interaction p-values indicate consistency across strata, whereas significant p-values suggest potential subgroup-specific effects.

All statistical analyses were performed using SPSS version 26.0 and R version 4.3.3, with forest plots generated utilizing GraphPad Prism version 10.0. A two-tailed p-value of less than 0.05 was deemed statistically significant.

## Result

### Baseline characteristics

To enhance the representativeness of the study population, **Table 1** provides baseline data from the MMC database for both study participants and excluded individuals, the latter defined as non-type 2 diabetic patients (N=4861). The results indicated that study participants exhibited higher age, AIP, BMI, FBG, and HbA1c levels, along with lower HDL-C levels, in comparison to the excluded individuals, which aligns with findings from prior studies. Therefore, it can be reasonably inferred that the participants included in this study are, to some extent, representative of the broader population.

**Table 1:** Baseline characteristics of study participants and exclusions.

Variables	Participants	Excluded persons	P
N	3094	4861	
Age, years	56.09 ± 10.64	54.37 ± 10.15	<0.001
Male (%)	1645 (53.16)	2783 (57.25)	<0.001
AIP	0.24 ± 0.36	0.03 ± 0.15	<0.001
BMI, kg/m^2^	24.67 ± 3.62	23.78 ± 3.33	<0.001
WC,cm	86.44 ± 10.11	80.35 ± 8.35	<0.001
DBP, mmHg	79.12 ± 11.40	77.41 ± 11.51	<0.001
SBP, mmHg	135.17 ± 20.80	126.81 ± 20.77	<0.001
LDL-C, mmol/L	2.85 ± 1.07	2.57 ± 0.83	<0.001
HDL-C, mmol/L	1.16 ± 0.36	1.55 ± 0.35	<0.001
TC, mmol/L	4.83 ± 1.91	2.57 ± 1.24	<0.001
TG, mmol/L	2.47 ± 2.49	2.17 ± 1.14	<0.001
Cr, μmol/L	78.23 ± 33.21	65.07 ± 22.06	<0.001
FBG, mmol/L	9.24 ± 3.40	5.85 ± 1.64	<0.001
HbA1c, %	9.76 ± 2.67	4.7 ± 1.07	<0.001
Current smoker	905 (29.25)	915 (18.82)	<0.001
Current drinker	959 (31.00)	987 (20.30)	<0.001
History of hypertension	1124 (36.32)	1542 (31.7)	<0.001

Data are summarized as number (percentage), mean ± standard deviation. AIP, Atherogenic index of plasma; BMI, body mass index; WC, waist circumference; DBP, diastolic blood pressure; SBP, systolic blood pressure; LDL-C, low-density lipoprotein cholesterol; HDL-C, high-density lipoprotein cholesterol; TC, total cholesterol; TG, triglyceride; Cr, creatinine; FBG, fasting blood glucose; HbA1c, glycated haemoglobin.

**Table 2** displays the baseline characteristics of patients categorized by their AIP tertiles (Q1: <0.07, Q2: 0.07-0.36, Q3: >0.36). This study encompassed a total of 3,094 patients with type 2 diabetes, with a mean age of 56.09 ± 10.64 years, of whom 53.16% were male. At present, smokers comprised 29.25%, and drinkers constituted 31.00%, while patients with a history of hypertension accounted for 36.32%. In comparison to the Q1 group, patients in the Q3 group were relatively younger, more likely to be male, and exhibited higher tendencies to smoke, consume alcohol, and have a history of hypertension. Furthermore, patients in higher AIP tertiles exhibited relatively elevated levels of BMI, WC, DBP, LDL, TC, TG, FBG, and HbA1c, while HDL-C levels were comparatively lower (All p < 0.05).

**Table 2:** Baseline characteristics of participants by AIP tertile.

Variables	Tatal	Q1(<0.07)	Q2(0.07-0.36)	Q3(>0.36)	P
N	3094	1032	1031	1031	
Age, years	56.09 ± 10.64	57.64 ± 9.84	56.72 ± 10.28	56.09 ± 10.64	<0.001
Male (%)	1645(53.16)	478(46.32)	541(52.47)	626(60.72)	<0.001
BMI, kg/m^2^	24.67 ± 3.62	23.40 ± 3.38	24.93 ± 3.54	25.68 ± 3.55	<0.001
WC,cm	86.44 ± 10.11	82.21 ± 9.79	87.33 ± 9.72	89.71 ± 9.36	<0.001
DBP, mmHg	79.12 ± 11.40	77.56 ± 11.27	79.61 ± 11.12	80.21 ± 11.65	<0.001
SBP, mmHg	135.17 ± 20.80	134.22 ± 21.04	136.17 ± 20.62	135.14 ± 20.71	0.11
LDL-C, mmol/L	2.85 ± 1.07	2.75 ± 1.04	2.77 ± 1.04	2.78 ± 1.10	<0.001
HDL-C, mmol/L	1.16 ± 0.36	1.43 ± 0.38	1.11 ± 0.24	0.93 ± 0.22	<0.001
TC, mmol/L	4.83 ± 1.91	4.55 ± 1.20	4.69 ± 1.22	5.27 ± 2.79	<0.001
TG, mmol/L	2.47 ± 2.49	1.07 ± 0.33	1.82 ± 0.50	4.51 ± 3.43	<0.001
Cr, μmol/L	78.23 ± 33.21	81.51 ± 47.29	69.73 ± 39.47	83.44 ± 32.49	0.60
FBG, mmol/L	9.24 ± 3.40	8.75 ± 3.08	9.21 ± 3.42	9.75 ± 3.62	<0.001
HbA1c, %	9.76 ± 2.67	9.42 ± 2.87	9.85 ± 2.57	10.01 ± 2.54	<0.001
Current smoker	905(29.25)	223(21.61)	304(29.49)	378(36.67)	<0.001
Current drinker	959(31.00)	258(25.00)	306(29.68)	395(38.31)	<0.001
History of hypertension	1124(36.32)	325(31.49)	397(38.51)	402(38.99)	<0.001

Data are summarized as number (percentage), mean ± standard deviation. AIP, Atherogenic index of plasma; BMI, body mass index; WC, waist circumference; DBP, diastolic blood pressure; SBP, systolic blood pressure; LDL-C, low-density lipoprotein cholesterol; HDL-C, high-density lipoprotein cholesterol; TC, total cholesterol; TG, triglyceride; Cr, creatinine; FBG, fasting blood glucose; HbA1c, glycated haemoglobin.

### Association of AIP with type 2 diabetic complications

**Figure 2:**
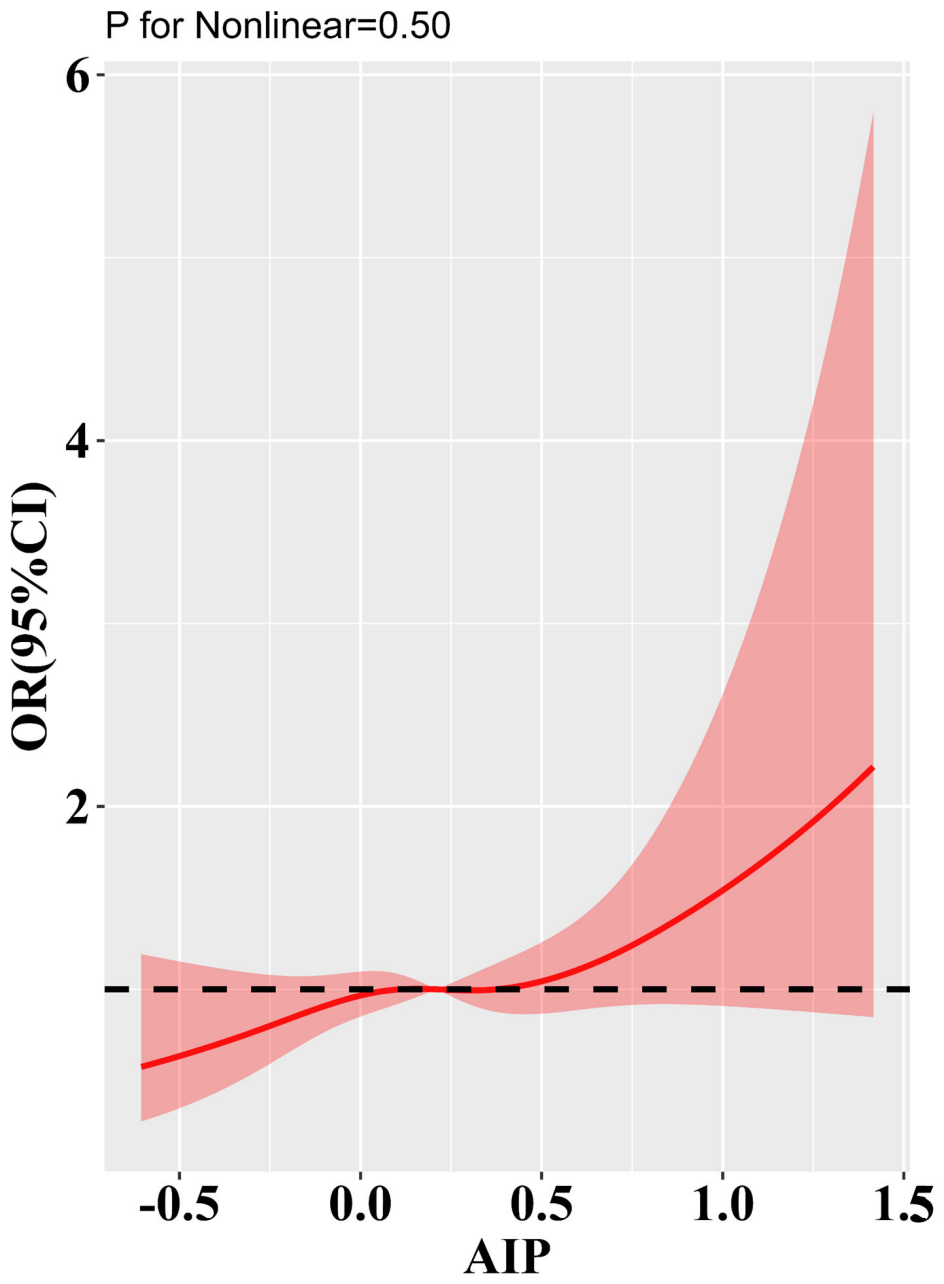
Results of RCS analysis of the a association of AIP with type 2 diabetic complications. Adjusted for age, sex, BMI, FBG, DBP, SBP, LDL-C, TC, FBG, HbA1c, smoking status, alcohol consumption, and history of hypertension.

**Table 3** presents the results of the logistic regression analysis. This study included a total of 1,653 cases of DA (53.4%), 676 cases of DKD (21.8%), 486 cases of DR (15.7%), and 1,149 cases of DPN (37.1%). When AIP was analyzed as a continuous variable, the logistic regression results indicated a significant association between AIP and DKD in all three adjusted models. In the fully adjusted model, the risk of developing DKD increased by 49% for each standard deviation increase in AIP. Notably, in Model 1, there appeared to be an inverse association between AIP levels and the risk of DPN; however, this association disappeared after further adjustment. When AIP was analyzed as a categorical variable based on tertiles, the association between AIP and DKD risk remained significant across all three adjusted models. In the fully adjusted model, the risk of DKD in the Q3 group was 22% higher compared to the Q1 group. Trend analysis further indicated that higher tertile groups corresponded to increased DKD risk (trend P < 0.05). Unfortunately, we observed no potential associations between AIP and other type 2 diabetic complications. **Figure 2** displays the results of the restricted cubic spline analysis. In the fully adjusted model, a clear dose-response relationship was observed between AIP and DKD risk (non-linear P = 0.50), with DKD risk starting to significantly increase when AIP > 0.41.

**Table 3:** Odds ratios and 95% CIs for the association of AIP with type 2 diabetic complications.

Variables	N	Q1	Q2	Q3	P	Per SD	P for trend
DA(N=1653)	Model 1	Reference	1.21(0.96,1.51)	1.15(0.92,1.45)	0.24	1.11(0.86,1.45)	0.23
	Model 2	Reference	1.03(0.79,1.33)	1.10(0.84,1.45)	0.35	1.20(0.86,1.68)	0.49
	Model 3	Reference	1.00(0.77,1.29)	1.05(0.80,1.39)	0.48	1.14(0.81,1.60)	0.71
DKD(N=676)	Model 1	Reference	1.53(1.22,1.92)	1.54(1.23,1.94)	<0.001	1.77(1.38,2.27)	<0.001
	Model 2	Reference	1.23(1.02,1.52)	1.21(1.01,1.51)	0.02	1.46(1.02,2.10)	0.02
	Model 3	Reference	1.23(1.03,1.54)	1,22(1.02,1.49)	0.01	1.49(1.03,2.15)	0.02
DR(N=486)	Model 1	Reference	0.91(0.70,1.18)	0.94(0.72,1.23)	0.77	0.94(0.69,1.28)	0.65
	Model 2	Reference	0.83(0.62,1.12)	0.81(0.59,1.12)	0.65	0.76(0.51,1.12)	0.19
	Model 3	Reference	0.83(0.61,1.11)	0.81(0.59,1.11)	0.66	0.75(0.51,1.11)	0.18
DPN(N=1149)	Model 1	Reference	1.02(0.85,1.23)	0.87(0.72,1.05)	0.17	0.79(0.64,0.98)	0.14
	Model 2	Reference	0.91(0.73,1.13)	0.84(0.67,1.06)	0.35	0.77(0.59,1.03)	0.15
	Model 3	Reference	0.91(0.73,1.13)	0.85(0.67,1.08)	0.28	0.78(0.59,1.04)	0.18

Model 1: adjusted for age and sex;

Model 2: adjusted for age, sex, BMI, WC, FBG, DBP, SBP, LDL-C, TC, HbA1c;

Model 3: Fully adjusted models, adjusted for age, sex, BMI, FBG, DBP, SBP, LDL-C, TC, FBG, HbA1c, smoking status, alcohol status, and history of hypertension.

AIP, Atherogenic index of plasma; BMI, body mass index; WC, waist circumference; FBG, fasting blood glucose; Per SD, odds ratio for per SD change in AIP.

### Subgroup analyses

To better elucidate the potential association between the AIP and DKD, we conducted subgroup analyses stratified by age, gender, BMI, and history of hypertension (**Table 4**). The results indicate that in each subgroup, the level of AIP consistently correlated with the risk of DKD. Furthermore, no significant interaction was observed between AIP levels and the subgroup variables (P for interaction > 0.05).

**Table 4:** Subgroup analyses for the association of the AIP with DKD.

Variables	OR (95% CI)
Age	
<60	1.40 (1.04,2.22)
≥60	1.97 (1.07,3.53)
P for interaction	0.51
Sex	
Male	1.59 (1.05,2.14)
Female	1.54 (1.03,2.09)
P for interaction	0.48
BMI	
<25	1.63 (1.01,2.68)
≥25	1.78 (1.21,2.63)
P for interaction	0.81
History of hypertension	
Yes	1.64 (1.08,2.51)
No	1.71 (1.25,2.34)
P for interaction	0.84

Adjusted for age, sex, DBP, SBP, LDL-C, TC, Cr, FBG, HbA1c, smoking status, alcohol status, and history of hypertension. BMI, body mass index; AIP, Atherogenic index of plasma; BMI, body mass index; WC, waist circumference; DKD, Type 2 diabetes combined with kidney damage; OR, odds ratio for per SD change in AIP.

## Discussion

In this retrospective cross-sectional study encompassing 3,094 patients with type 2 diabetes from China, we identified a persistent positive correlation between the AIP and the risk of DKD, which remained statistically significant after comprehensive adjustments for various confounding factors. Subgroup analyses further corroborated the robustness of this association, illustrating its generalizability across diverse subgroup populations. Moreover, the RCS analysis indicated a potential dose-response relationship between AIP and DKD. These findings suggest that AIP may function as an early biomarker for predicting DKD.

Type 2 diabetes is widely recognized as a chronic disease characterized by its gradual onset and progressive nature (28). Despite the development of various medications for the treatment of type 2 diabetes, effective curative methods remain insufficiently addressed (29). Consequently, upon diagnosis, type 2 diabetes can exert lifelong impacts on patients, particularly manifesting as a spectrum of multisystem complications, including circulatory and neurological damage, which can severely compromise patients’ quality of life and impose substantial economic burdens on both individuals and the healthcare system (30, 31). Recent studies increasingly indicate an alarming rise in the incidence of type 2 diabetes among younger populations, consequently leading to an increase isability-adjusted life years associated with the condition (32). Encouragingly, recent research underscores that the adoption of proactive prevention and treatment strategies can substantially delay the onset and progression of type 2 diabetes and its associated complications, thereby reducing healthcare costs and enhancing patients’ quality of life (33, 34). With recent advancements in high-throughput testing technologies, an increasing number of researchers are exploring biomarkers for the early prediction of type 2 diabetes and its associated systemic complications through methodologies such as metabolomics and proteomics (35, 36). However, the limited sample sizes in the corresponding observational studies have hindered the translation of these findings into clinical applications.

Recent studies have identified potential associations between individual plasma lipid components, such as LDL-C and HDL-C, and type 2 diabetic complications; however, reliance solely on these individual components may hinder a comprehensive assessment of systemic damage in affected patients (37, 38). Consequently, numerous studies have sought to propose various composite indicators, such as the glucose-triglyceride index, to enhance the prediction of disease onset and progression (39). In contrast to individual lipid components, the AIP serves as a more comprehensive indicator, derived from plasma lipid profiling. Existing research suggests that AIP correlates with lipoprotein particle size and reflects the interplay between atheroprotective and atherogenic particles (40). The National Cholesterol Education Program (NCEP) in the United States regards AIP as a critical marker of plasma atherogenicity and recognizes it as a reliable indicator for predicting cardiovascular risk (41). A longitudinal study conducted within the China Health and Retirement Longitudinal Study, which included 8,760 participants, revealed that alterations in the AIP from baseline to follow-up can serve as predictors for the risk of developing type 2 diabetes. Specifically, individuals exhibiting persistently high AIP levels, as well as those whose AIP fluctuated between high and low, experienced an approximately 1.5-fold increased risk of developing type 2 diabetes compared to individuals with persistently low AIP levels (42). A meta-analysis further suggested that AIP serves as a direct and reliable biomarker for assessing the risk of developing type 2 diabetes (43). These studies underscore the potential association between AIP and both the onset and progression of type 2 diabetes. Presently, research investigating the association between AIP and type 2 diabetic complications remains relatively limited. A study conducted by Li et al. (44) revealed that in patients with type 2 diabetes, AIP was linked to the risk of hypertension; individuals with elevated AIP levels exhibited a significantly increased prevalence of DPN and metabolic syndrome. Another cohort study identified a potential association between AIP and the risk of DKD, which corroborates the findings of our study (45).

We hypothesize that the association between the AIP and DKD may be elucidated through several potential mechanisms. Firstly, AIP serves as a marker of plasma lipoprotein metabolism and demonstrates a positive correlation with small dense low-density lipoprotein (sdLDL). It is well established that sdLDL serves as a predictor of atherosclerosis owing to its small size, low plasma clearance rate, and heightened sensitivity to oxidative stress, which can provoke subendothelial inflammation (46). The onset and progression of DKD undoubtedly involve the intricate interplay of various factors, including oxidative stress and endothelial dysfunction (47). Secondly, the development of DKD is distinctly associated with decreased levels of HDL-C and elevated TG (48). In the context of type 2 diabetes, the potential protective functions of HDL-C—including reverse cholesterol transport as well as its antioxidant and anti-inflammatory roles—may be significantly compromised (49). Nevertheless, the specific mechanisms by which AIP influences type 2 diabetic complications remain unclear and necessitate further investigation through both basic and clinical studies.

This study presents several notable advantages. Firstly, our sample and corresponding data were derived from the MMC platform, which adheres to stringent management standards, thereby ensuring the reliability of the findings. Secondly, the implementation of RCS analysis and subgroup analyses bolstered the statistical power and validated the robustness of our findings. Nonetheless, this study is not without several limitations. Firstly, this research design is a cross-sectional observational study, which does not facilitate the establishment of clear causal relationships. Secondly, unaccounted variables—including dietary patterns, ethnic differences, and lifestyle factors—may introduce bias into the results. Furthermore, the type 2 diabetes patients in this study predominantly originated from China, necessitating caution when generalizing the results to other ethnic groups. Thirdly, the absence of detailed information regarding the participants’ medical history and medication usage may introduce bias into the results. To address this limitation, the research team plans to collect additional data in future studies, aiming to establish a more comprehensive and high-quality clinical research database. Finally, variables such as LDL-C and TC may introduce multicollinearity with AIP, potentially leading to biased results. However, given that these variables are commonly used clinical markers and are associated with a wide range of diseases, combined with the relatively large sample size of the current study, the potential bias arising from this issue is likely to be minimal.

## Conclusions

In conclusion, elevated AIP levels are significantly correlated with an increased risk of DKD, with evidence suggesting a potential dose-response relationship between AIP levels and DKD risk. These findings indicate that AIP could function as a valuable predictive biomarker for evaluating the risk of renal injury in patients with type 2 diabetes. Regular screening of AIP levels in patients with type 2 diabetes may provide significant benefits in the prevention of DKD.

## Data Availability

The raw data supporting the conclusions of this article will be made available by the authors, without undue reservation.
